# Rater severity differences in English language as a second language speaking assessment based on rating experience, training experience, and teaching experience through many-faceted Rasch measurement analysis

**DOI:** 10.3389/fpsyg.2022.941084

**Published:** 2022-07-22

**Authors:** Muhamad Firdaus Mohd Noh, Mohd Effendi Ewan Mohd Matore

**Affiliations:** ^1^Sekolah Rendah Agama Bersepadu Segamat, Johor, Malaysia; ^2^Research Centre of Education Leadership and Policy, Faculty of Education, Universiti Kebangsaan Malaysia (UKM), Selangor, Malaysia

**Keywords:** severity, rater, speaking, teachers’ experience, training experience, teaching experience, MFRM, differences

## Abstract

Evaluating candidates’ answers in speaking skill is difficult and rarely explored. This task is challenging and can bring inconsistency in the rating quality among raters, especially in speaking assessments. Severe raters will bring more harm than good to the results that candidates receive. Many-faceted Rasch measurement (MFRM) was used to explore the differences in teachers’ rating severity based on their rating experience, training experience, and teaching experience. The research uses a quantitative approach and a survey method to enlist 164 English teachers who teach lower secondary school pupils, who were chosen through a multistage clustered sampling procedure. All the facets involving teachers, candidates, items, and domains were calibrated using MFRM. Every teacher scored six candidates’ responses in a speaking test consisting of three question items, and they were evaluated across three domains, namely vocabulary, grammar, and communicative competence. Results highlight that the rating quality was different in terms of teachers’ rating experience and teaching experience. However, training experience did not bring any difference to teachers’ rating quality on speaking test. The evidence from this study suggests that the two main factors of teaching and rating experience must be considered when appointing raters for the speaking test. The quality of training must be improved to produce a rater with good professional judgment. Raters need to be supplied with answer samples with varied levels of candidates’ performance to practice before becoming a good rater. Further research might explore any other rater bias that may impact the psychological well-being of certain groups of students.

## Introduction

Speaking assessment is an integral part of language testing that aims at distinguishing candidates’ capabilities in using the targeted language through spoken production. Candidates’ capabilities are appraised on the repertoire of domains in speaking skills, such as fluency, accuracy, vocabulary, enunciation, grammar, accentedness, and comprehensibility (Namaziandost, 2019). These domains are selected based on the demands of the assessment and the types of items used. Interviews, storytelling, and discussions are among the common types of items normally used in speaking test. The execution of a speaking test is typically involving an interlocutor to interact with candidates and employs human raters to evaluate candidates’ spoken production by giving marks based on the marking scales (Sheetz et al., 2018). A substantial consideration in the scoring process of speaking assessment is rating quality produced by raters (Fan and Yan, 2020). Rating quality is a fundamental property of a valid and reliable assessment system (Aera, 2014). It has long been a debating topic among scholars and researchers in educational assessment as it is the main factor in ascertaining whether candidates are evaluated with fairness, reliability, objectivity, and validity principles. These principles may be threatened due to the existence of variability among raters in terms of many factors (Fan and Yan, 2020). The factors include rating experience, teaching experience, gender, training experience, familiarity with candidates, language proficiency, and level of education (Eckes, 2015). These variations associated with raters’ characteristics may obscure the assessed constructs or domains, contribute to rating errors, and eventually give a wrong interpretation of candidates’ actual capabilities. Rating errors attributable to raters’ variability must be mitigated, especially in high-stakes assessments and large-scale settings, because the results can impact the candidates’ future. Variability among raters is central to the rating process of speaking assessment because each rater brings their own idiosyncrasies and values to the rating scene including their experiences (Engelhard and Wind, 2018). Raters’ experiences have been examined in previous studies by other researchers but are still underexplored, especially in terms of speaking assessment (Han, 2016; Fan and Knoch, 2019). The studies investigating the effect of raters’ rating experiences have resulted in contradictory findings. Alp et al. (2018), Ahmadi Shirazi (2019), and Park (2020) have reported that raters’ experiences did not lead to significantly different rating quality among raters. However, other studies have concurred that different rating performances were observed when raters with different experiences rated the same candidates (Kim and Lee, 2015; Attali, 2016; Davis, 2016; Huang et al., 2018; Park, 2020). Raters’ experiences were investigated in terms of their rating experience, teaching experience, and also their experience in attending assessment training. Importantly, those studies lead to inconsistent findings, and no solid conclusion can be made about whether raters’ experience can affect rating quality.

Thus, this study will shed some light on whether raters’ severity level in assessing speaking test can be interrupted when they have different background experience. To this end, this study delved into investigating whether raters’ rating quality is affected by their different level of experiences (teaching, rating, and training). Therefore, this study has three leading objectives:

1. To determine the differences in severity among teachers with different rating experience.
2. To determine the differences in severity among teachers with different training experience.
3. To determine the differences in severity among teachers with different teaching experience.

Hence, the following hypotheses are constructed:

H1: There is no significant difference in severity among teachers of different rating experience.
H2: There is no significant difference in severity among teachers of different training experience.
H3: There is no significant difference in severity among teachers of different teaching experience.

## Literature review

### Rating process

A good assessment system is a well-planned procedure that begins with the construction and selection of items for assessment administration, then to the rating and marking process before results are used in determining candidates’ future (Gerritsen-van Leeuwenkamp et al., 2017). Each of these stages is crucial, especially during the rating process, because one’s result depends on the extent to which the rating is executed in accordance to sound psychometric quality. Rating refers to the process of awarding evaluation code in the form of marks, and grade of statements about candidates’ performance in particular skills or domains (Cohen-Swerdlik, 2009). This process entails marking, judging, and evaluating candidates’ answers based on consensual standards set by an appointed body. The rating process is much affected by the types of questions chosen in the assessment (Eckes, 2015). Objective questions, also known as selected-response items, are much easier to be marked as the answer schemes are provided. The marking style is known as dichotomous marking during which candidates get marks when they are able to provide the same answers with the answer schemes, but they do not receive any mark if they write different answers (McKenna, 2019). Meanwhile, subjective questions, also known as constructed-response items, are more liberal in accepting candidates’ answers. The marking scheme or rubrics are provided merely to guide guidance for raters on how to mark but not to limit the accepted answers (Albano and Rodrigues, 2018). From the psychometric perspective, this type of question intends to assess candidates in a more sophisticated and complex skills that may entail many sub-domains (Zlatkin-Troitschanskaia et al., 2019).

A significant difference between the rating process of objective and subjective items is the amount of freedom granted to raters when awarding marks to candidates’ answers (Tomas et al., 2019). While raters enjoy the liberty to accept or reject candidates’ answers when rating subjective items, there is also the probability of construct-irrelevance variance introduced by raters. Bond and Fox (2015) highlighted that the variance gives a negative impact on the estimates of candidates’ competency measures. However, in an operational assessment setting, having only one rater to rate answers of all candidates to avoid construct-irrelevance variance is costly and far from practical (Jones and Wind, 2018). Due to the same reason, it is also impossible to assign all raters to rate the answers of all candidates. Consequently, this situation leads to the existence of variability among raters that may interrupt the production of truly valid and reliable scores that can generate a wrong conclusion about candidates’ competency level (Wu and Tan, 2016). Ideally, all the appointed raters should share the same understanding of how rating should be done. Also, they need to prove that they have a mutual interpretation of items, domains, and rubrics (Engelhard and Wind, 2018). However, it is not an easy task because each rater has different backgrounds and experiences that can affect the way they rate candidates’ answers (Lamprianou, 2018). It is even more challenging in speaking assessment because candidates’ answers are normally not recorded, and the rating process is executed the moment candidates produce the answers.

### Raters’ experience

The rating process happens when teachers as raters interact with candidates, listen to the candidates’ answer, and then decide on one score to summarize the candidate’s performance in particular skill domains. Throughout the process, a teacher does not only use their professional judgment but also their background. Literature has widely reported that one’s background can either positively or negatively impact how one rates candidates. Raters’ backgrounds include rating experience (Huang et al., 2018; Ahmadi Shirazi, 2019; Şahan and Razı, 2020), training experience (Duijm et al., 2017; Bijani, 2018, 2019), teaching experience (Kang and Veitch, 2017; Eckstein and Univer, 2018; Kang et al., 2019), raters’ first language (Hijikata-Someya et al., 2015; Marefat and Heydari, 2016; Ahmadi Shirazi, 2019; Kang et al., 2019), familiarity about candidates (Huang et al., 2016; Tanriverdi-Koksal and Ortactepe, 2017; Wikse Barrow et al., 2019), personal traits, gender (Bijani and Khabiri, 2017; Protivínský and Münich, 2018), academic achievement (He et al., 2013; Soltero-González et al., 2016), age (Soltero-González et al., 2016; Isbell, 2017), and cultural background (Stassenko et al., 2014). In terms of raters’ experiences, three types of experience are widely examined, which are rating experience, training experience, and teaching experience. Rating experience refers to the experience that a rater has in any scoring procedures in a defined assessment setting. Ideally, with more rating experience, raters’ rating quality would be better (Eckes, 2015). Studies on how rating experience can affect raters’ ratings have employed many different groups of raters as respondents who are divided into groups based on research contexts (Duijm et al., 2017; Kang and Veitch, 2017; Bijani, 2018, 2019; Eckstein and Univer, 2018; Huang et al., 2018; Ahmadi Shirazi, 2019; Kang et al., 2019; Şahan and Razı, 2020). Findings from the research have mainly reported that a significant difference was discovered among the rater groups.

When a significant difference was observed, new raters tended to portray more variability in the ratings they generated in comparison to experienced raters (Lim, 2011; Kim and Lee, 2015). In other words, new raters were not consistent as some of them were too severe, while some other new raters were too lenient (Lim, 2011). Erratic ratings were also observed among new raters rendering them inconsistent as a rater (Kim and Lee, 2015). On the other hand, with more rating experience, experienced raters were discovered to use a high level of severity because they develop critical and analytical cognition (Barkaoui, 2010a). Also, they tended to use other criteria than what is prescribed in the rubrics and gave more attention to language accuracy, and also provided longer qualitative comments (Barkaoui, 2010a; Leckie and Baird, 2011). However, some other studies have also reported that both groups of raters did not manifest a uniform rating and the rating pattern was not obvious (Ahmadi Shirazi, 2019; Sahan and Razi, 2020).

The purpose of rater training is to empower participants to become a quality rater. The objective of rater training is achieved when raters manage to rate candidates’ answers without construct-irrelevance variance and when they do not use factors other than candidates’ answers when finalizing marks for candidates. The literature has reported consistent findings examining the differences in rating quality caused by raters’ different amount of training experience (Tajeddin and Alemi, 2014; Davis, 2016; Seker, 2018). Raters showed improvement in the rating quality after they successfully attended training (Tajeddin and Alemi, 2014; Davis, 2016; Seker, 2018). They also managed to reduce their dispersion index among them, which indicates that their ratings were homogenous (Tajeddin and Alemi, 2014). In terms of severity level, raters who have attended training showed that they were able to produce ratings that are closer to the mean score, which is desirable in any operational scoring (Kang et al., 2019). However, studies have also reported that training is more influential to novice raters as compared to experienced raters (Kim and Lee, 2015). Interestingly, a difference was also observed when comparing raters based on the times they attended the training. Raters who have just attended training show more stable ratings as compared to those who attended the training a long time.

As most studies were carried out in educational settings, teaching experience is also included in the discussion. Length of tenure as a teacher is claimed to be a confounding factor in the quality of raters’ ratings (Park, 2020). It was reported that new teachers rated with leniency because they tended to give high marks consistently to candidates, while experienced teachers were severe as they preferred to penalize every single detail of sub-skills, such as grammar (Lee, 2016). Similar findings were also concluded (Tsunemoto et al., 2020) that raters with extensive teaching experience put high expectation on the candidates’ accuracy. These teachers showed high severity, especially when assessing candidates’ pronunciation accuracy as compared to raters with less experience who rate candidates with leniency. Interestingly, through qualitative data collection, raters also admit that their rating quality is much influenced by their personal teaching experience rather than the provided scoring rubric (Huang et al., 2020). The discussion about teaching experience has also included the assessment domains that raters focus on (Huang, 2013; Eckstein and Univer, 2018; Kang et al., 2019). For instance, native speaker teachers who have experience in teaching English as a first language put a high value on originality and criticality of students’ work, while non-native teachers with experience in teaching English as a second language were inclined to prioritize lexical and grammatical features of students’ text (Eckstein and Univer, 2018; Kang et al., 2019). In other studies, raters with no teaching experience were better at discriminating candidates’ answers according to distinct linguistic domains (Hsieh, 2011; Huang, 2013) and were not interrupted by candidates’ foreign accents (Huang, 2013).

## Materials and methods

### Samples

A total of 164 English teachers were selected as respondents of the study through multistage clustered sampling from the Selangor district in Malaysia. All the teachers are teaching lower secondary school students (from one, two, and three) in preparation for the final examination in form three, namely *Pentaksiran Tingkatan Tiga* (PT3). The respondents completed a background questionnaire about their experience. As shown in Table 1, they varied in their professional experience in rating speaking test, teaching the English language as a second language (ESL), and attendance in rater training. In terms of rating experience, the first group of respondents (63 teachers) had no experience in rating speaking test in PT3. The second group of 44 teachers self-reported having 1–3 years of PT3 rating experience and the third group of 57 teachers reported having 4–6 years of experience in rating PT3. Regarding attendance to rater training, 102 teachers have attended rater training. Only training on language high-stake assessment scoring such as PT3 was considered valid to acknowledge the teachers have attended the training. According to the years of teaching experience, 50 teachers self-reported that they have been teaching ESL for 1–10 years, 56 teachers identified themselves as having 11–20 years of teaching experience, while the remaining 58 claimed that they have been teaching for more than 20 years.

**TABLE 1:** Respondents’ profile.

Experiences	Number of teachers	Percentage (%)
Rating experience		
No experience	63	38.4
1–3 years of experience	44	26.8
4–6 years of experience	57	34.8
Rater training experience		
Have attended	102	62.2
Never attended	62	37.8
Teaching experience		
1–10 years of experience	50	30.5
11–20 years of experience	56	34.1
More than 20 years of experience	58	35.4

### Instrumentation

The main instrument in this study is candidates’ answer samples in a speaking test, which was recorded and validated by five panels of experts. The process of producing the instruments began with validating the item questions, recording the candidates’ answers, and then validating the recording. Three question items constituted the speaking test conducted in this study, and they are background interview (Item 1), storytelling (Item 2), and discussion (Item 3). In Item 1, candidates were asked about their names, personal opinions about English Language learning, hobbies, and activities they enjoyed with their friends. As for Item 2, candidates were given five pictures of a scenario of people going for a picnic at the beach, and they were asked to tell a story. Finally, for Item 3, candidates were provided with a bubble map about what students should bring when going on a jungle trekking. Six points were given, and they needed to discuss with their partner which item was the most important one.

The validation of the three items with the expert panels was calculated using the Content Validity Ratio (CVR), and all the panels agreed that the three items were suitable for the study. Next, a total of 30 lower secondary school students were assigned to answer the test, and their answers were recorded. They are of different genders (10 males and 20 females) and ethnicity (16 Malay, 10 Indian, and 4 Chinese). Based on their recent examination result, the candidates are heterogenous in their language proficiency. They self-reported their recent grade in English Language subject (6 candidates received an A, 6 candidates received a B, 10 candidates received a C, and 8 candidates received a D). To carry out the speaking test, an interlocutor was tasked to ask the candidates the questions. The first and second items were individual, during which the interlocutor interacted with the candidates. They were assessed based on two domains in the first two items: vocabulary and grammar. Then, Item 3 was carried out in pair, during which candidates needed to interact with their peer based on the situation described by the interlocutor and the information aided on a bubble map. The candidates were assessed based on three domains: vocabulary, grammar, and communicative competence. The raters gave different marks to each individual candidate based on their interaction. The test used individual and interactional question items because both types offer distinct benefits and rich information about candidates’ abilities as well as manage to capture well raters’ capability to score candidates (Sheetz et al., 2018). Apart from the recording of candidates’ answers, the raters were also provided with a rating rubric and scoring sheet. The rubric consisted of three skill domains that the teachers need to focus on which are vocabulary, grammar, and communicative competence. The rubric was also validated by the same expert panels, and they all agree that the rubric is suitable to be used in the study.

### Rating collection

The analytical rubric was used because it can delineate candidates’ sub-skills in speaking skill and require teachers to manifest their expertise to score with good quality (Yamanishi et al., 2019). Each mark provided by the teachers for the three domain is useful for analysis of the teachers’ rating quality (Badia, 2019). The ratings from teachers were collected using a linked rating design that is suitable to be used when teachers could not rate all the candidates in the assessment (Jones and Wind, 2018). A systematic rating system mapping was established to ensure that enough link is created between teacher, candidate, item, and domain facets to enable analysis using many-faceted Rasch measurement (MFRM). Through the systematic rating system mapping, each rater only needed to rate six candidates, and each candidate was rated by 10 raters. Altogether, a total of 6,886 score units were generated.

## Results

### Assumptions of the Rasch model

**FIGURE 1:**
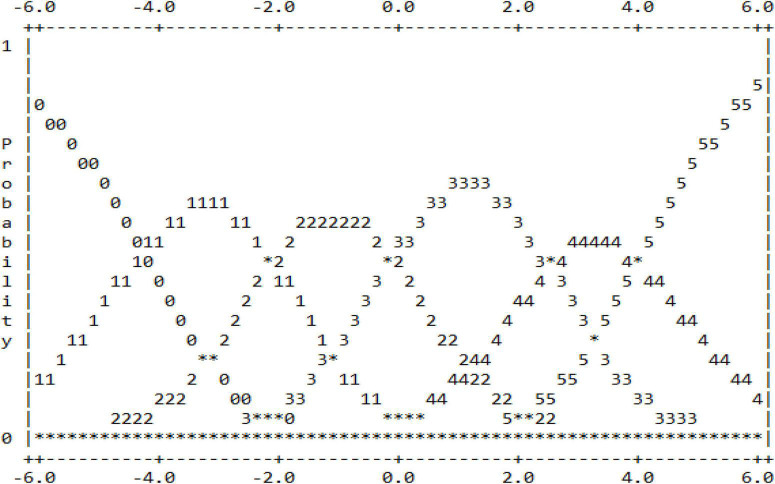
Scale threshold values.

Before data analysis, data preparation and statistical assumptions need to be made to ensure that the data collected is suitable for analysis (Bond and Fox, 2015). Data preparation begins with checking for missing data. The results of the descriptive analysis showed that there were no missing data. The data preparation process then included assuming item fit, item separation, and rating scale function. Table 2 shows the report of the fit for the three items used in the study to determine whether the items are suitable before further analysis is carried out. The infit MnSq values of all the three items are within the range of 1.00–1.01 logits, while the outfit MnSq values are within the range of 0.99–1.01. These values are still accepted because they fall within the range of 0.77–1.30 logits as outlined by Bond and Fox (2015). Whereas the Zstd values for all items are within −0.3 to 0.1 and are still under the acceptable range, ±2 as recommended by Bond and Fox (2015). Based on the values of infit and outfit, the three items are fit to be used in the analysis. Next, the report on item separation is needed to ensure to what extents the three items can discriminate candidates’ capabilities. Table 3 illustrates that the separation ratio is 9.01, which indicates that the difficulty of the items is separated into nine strata, while the separation index is 12.35, which means the items can discriminate the candidates into more than 12 strata based on their capabilities. Separation reliability hits 0.99, which means the item separation analysis is run based on valid measurement procedures. Finally, scale functioning analysis is run to examine the extent to which the scales used function to measure the constructs and the extent to which the raters use all the categorical scales, and their consistency in using the scales (Barkaoui, 2010b). Apart from that, it also reports on raters’ uniformity in interpreting the scales and providing evidence of central tendency incidents (Bond and Fox, 2015). Table 4 indicates the report on scale functioning, which is analyzed through six criteria (Bond and Fox, 2015). First, each category of scale must be awarded more than 10 times to candidates more (Bond and Fox, 2015). All the six scales in this study were reported to be used by raters more than 10 times, ranging from 86 (Scale 0) to 27,111 (Scale 3). Second, the average values for each scale need to be monotonical. This criterion is fulfilled as the average values for each scale is ascending systematically, starting from −2.12 logits for scale 0 to −1.58 logits (Scale 1), to 0.52 (Scale 2), to 0.66 (Scale 3), to 1.87 (Scale 4), and eventually to 2.94 for Scale 5. Third, the infit MnSq value must be less than 2.0 logits. This criterion is also fulfilled as the values for all the scale categories are ranged from 0.9 to 1.3. Next, the fourth criterion outlines that the threshold needs to be ascending. The report shows that the threshold starts with −4.27 and further increases to −2.1, −0.12, 2.51, and eventually 3.98. Next, the fifth criterion conditions that the scale threshold difference must be between the range of 1.0–5.0. If the values are less than 1.0, the scales need to be combined. Likewise, if the values are more than 5.0, the scales need to be split. Table 5 depicts that the threshold for all the scales fall under the accepted range. Finally, the last criterion points out that the curves for each scale must be visible and not hidden between one another. The existence of an invisible curve is problematic because it indicates that the scale category is not chosen by raters when rating candidates’ work. Figure 1 shows that the peak for each scale is clearly seen, and no scale is hidden behind another scale. All the six criteria are fulfilled in the study, and thus indicating that all the scales are valid to be used in the next analysis to answer the research questions.

**TABLE 2:** Report on item fit.

Item	Total score	Logits	SE	Infit		Outfit		Point measure	
				MnSq	Zstd	MnSq	Zstd	Correlation	Expected
Interview	5,195	−0.20	0.03	1.01	0.1	1.01	0.1	0.71	0.71
Story telling	5,120	−0.13	0.03	1.00	−0.1	0.99	−0.1	0.69	0.71
Discussion	7,126	0.33	0.03	1.00	−0.1	0.99	−0.3	0.73	0.71
Mean	5,813.7	0.00	0.03	1.00	0.0	1.00	−0.1	0.71	–
SD (population)	928.5	0.23	0.00	0.00	0.2	0.01	0.2	0.01	–
SD (samples)	1,137.1	0.29	0.00	0.01	0.2	0.01	0.3	0.02	–

**TABLE 3:** Report on item separation.

Statistics	Values
Separation ratio	9.01
Separation index	12.35
Separation reliability	0.99

**TABLE 4:** Report on scale functioning.

Data				Quality control		Outfit MnSq	Rasch-Andrich threshold		Expectation measure at		Most probable for	Rasch-Thurstone threshold	Category Peak probability (%)
Scale	Used	%	Cum. %	Average	Expected								
0	86	1	1	−2.12	−2.44	1.3	−	−	−5.42		Low	Low	100
1	807	12	13	−1.63	−1.58	0.9	−4.27	0.11	−3.22	−4.5	−4.27	−4.37	59
2	2,278	33	46	−0.52	−0.53	1	−2.1	0.04	−1.09	−2.13	−2.1	−2.11	57
3	2,711	40	86	0.66	0.66	1	−0.12	0.03	1.17	−0.01	−0.12	−0.07	64
4	785	11	98	1.87	1.87	1	2.51	0.04	3.28	2.3	2.51	2.39	51
5	161	2	100	2.94	2.89	1	3.98	0.09	−5.2	4.36	3.98	4.14	100

**TABLE 5:** Report on threshold changes.

Pair of scales	Gaps	Threshold
S_0–1_	0.00 to −4.27	1.00 < 4.27 < 5.00
S_1–2_	− 4.27 to −2.1	1.00 < 2.17 < 5.00
S_2–3_	−2.1 to −0.12	1.00 < 1.98 < 5.00
S_3–4_	− 0.21 to 2.51	1.00 < 2.72 < 5.00
S_4–5_	2.51 to 3.98	1.00 < 1.47 < 5.00

### Main effect analysis

**FIGURE 2:**
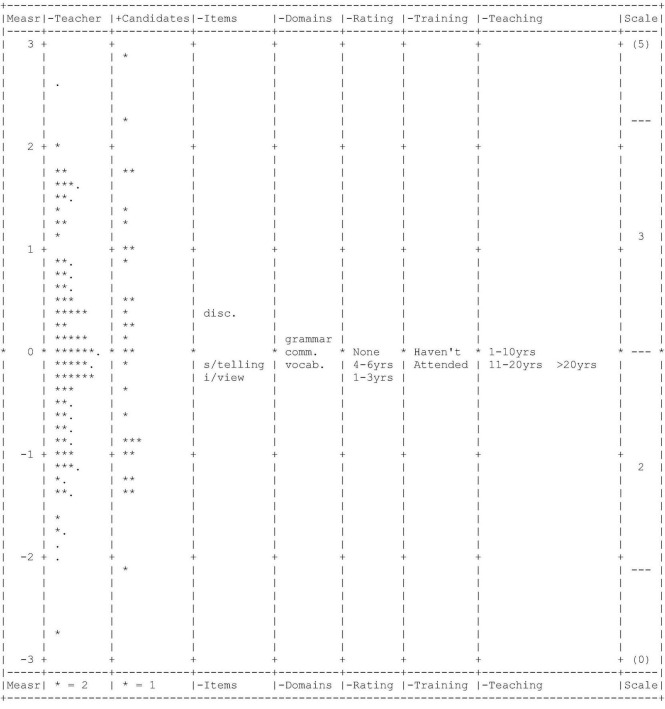
Wright map.

After the calibration of the main facets (teachers, candidates, items, and domains) with the other three dummy facets, FACETS reported the analysis of each main facet. Each unit of every facet was arranged according to its respective parameter in a graphical display, known as the Wright map, shown in Figure 2. The first column is a frame of reference for all the facets in the form of interval-logit scales from 3 to −3. The second column positions all the teachers according to their severity level, starting from the most severe teacher on top to the most lenient teacher at the bottom. Next, in the third column, the 30 candidates are arranged according to their ability level. Then, based on their levels of difficulty, the fourth column locates the three items, and the fifth column positions the three domains. The most difficult item is discussion, followed by storytelling and background interview. Whereas grammar precedes other domains as the most difficult, followed by vocabulary and communicative competence. Finally, the sixth, seventh, and eight columns situate the three dummy facets: rating experience, rater training, and teaching experience.

### The differences in severity among teachers of different rating experiences

The teachers were divided into three groups based on the number of years of their rating experience. The first group is teachers without experience in rating high-stakes assessments, while the second group comprises teachers with 1–3 years of experience in rating high-stakes assessments, and the teachers in the third group possess between 4 and 6 years of experience. The findings in Table 6 indicate that the third group of teachers manifested the highest severity level with 0.03 logits, followed by the second group (−0.06 logits), and finally the first group (−0.20 logits). It means that teachers with the highest years of rating experience were the most severe raters and teachers without any rating experience were the most lenient raters. The Chi-square analysis depicted that the differences in severity among the three groups were statistically significant, with the Chi-square value, χ^2^ = 26.0, *df* = 2, *p* < 0.01. Thus, the null hypothesis was rejected. Furthermore, the Chi-square test has shown that teachers in each group rated with different severity levels, with a *p*-value < 0.01, as presented in Table 6. The separation ratio for the first and second groups of teachers, as shown in Table 7, were, respectively, 4.35 and 4.21, indicating that their severity was four times higher than standard errors. Meanwhile, the value for the third group was 5.72, suggesting that their severity was more than five times bigger than standard errors. Next, the separation index informs the number of severity strata among the teachers within their respective groups. It was found that the third group were stratified into almost eight different severity groups, while the first and the second groups are divided into six severity strata. The separation reliability of all the groups managed to achieve high values, 0.95 and 0.97, indicating that the separation statistics provided for all the groups are highly reliable.

**TABLE 6:** Teachers’ differences in severity based on rating experience.

Group	Total score	Total count	Observed average	Measure	Model SE
No experience	5,062	1,837	2.8	−0.20	0.03
1–3 years of experience	6,077	2,384	2.5	−0.06	0.03
4–6 years of experience	6,312	2,607	2.4	0.03	0.03
Mean	5,813.7	2,276	2.6	−0.08	0.03
SD	670	396.2	0.2	0.12	0.00

Fixed (all same) Chi-square: 26.0, *df*: 2, significances (probability): 0.00.

**TABLE 7:** Rater facet report based on rating experience.

Group	No experience	1–3 years of experience	4–6 years of experience
χ^2^	1,265.9	828.1	1,916.6
*df*	62	43	56
Significance	0.00	0.00	0.00
Separation ratio	4.35	4.21	5.72
Separation index	6.13	6.02	7.96
Separation reliability	0.95	0.95	0.97

### The differences in severity among teachers of different training experience

To investigate the differences in severity based on teachers’ training experience, the teachers were divided into two groups: teachers with experience in rater training and teachers who never attended the training. The ratings they produced were then compared to answer the research question. Table 8 depicts the results of the analysis. The findings of the chi-square test showed that differences were not statistically significant, with the chi-square value, χ^2^ = 3.1, *df* = 1, *p* > 0.01. Thus, the null hypothesis failed to be rejected. However, teachers from both groups were rated with different severity levels. Teachers in the second group rated with a slightly higher severity level with −0.01 logits, as compared to teachers in the first group with −0.07 logits. Table 9 illustrates that the separation statistics appeared to be almost similar for both groups of teachers. The separation ratio for the first group was 4.18, while 4.96 for the second group, suggesting that both groups of teachers used four times bigger severity as compared to the standard errors regardless of their training background. Then, based on the separation index values, the first group of teachers was divided into five strata, while the second group of teachers was separated into six strata.

**TABLE 8:** Teachers’ differences in severity based on training experience.

Group	Total score	Total count	Observed average	Measure	Model SE
Have attended training	10,353	4,243	2.6	−0.07	0.02
Never attended training	6,488	2,585	2.5	−0.01	0.03
Mean	8,720.5	3,414	2.5	−0.04	0.03
SD	3,157.2	1,172.4	0.1	0.05	0.00

Model, fixed (all same) Chi-square: 3.1, *df*: 1, significance (probability): 0.08.

**TABLE 9:** Rater facet report based on training experience.

Group	Attended training	No training
χ^2^	1,878.7	1,625.3
*Df*	101	61
Significance	0.00	0.00
Separation ratio	4.18	4.96
Separation index	5.91	6.95
Separation reliability	0.95	0.96

### The differences in severity among teachers of different teaching experience

In terms of teaching experience, the teachers were divided into three groups. The first group was teachers who had 1–10 years of teaching experience, while the second group was teachers who had 11–20 years of teaching experience, and the third group was teachers who had more than 20 years of teaching experience. The rationale behind the interval of the teachers was that the teachers’ length of teaching experience ranged from 1 to 28 years. Therefore, dividing them into three groups was rather practical to best answer the question of whether there is any difference in their severity.

The result of the analysis is shown in Table 10. It was discovered that the third group of teachers scored the candidates with the highest level of severity with a logit value of 0.02, followed by the first group (−0.1 logits), and finally, the second group of teachers scored with the lowest severity level (−0.07 logits). The Chi-square analysis depicted that the differences in severity among the three groups were statistically significant, with the Chi-square value, χ^2^ = 7.3, *df* = 2, *p* < 0.01. Thus, the null hypothesis was rejected. Furthermore, the Chi-square test showed that teachers in each group rated with different severity levels, with *p*-value < 0.01, as presented in Table 11. The separation ratio for the first group was 4.64, 5.05 for the second group, and 8.18 for the third group, indicating that their severity was, respectively, four times, five times, and eight times bigger than the standard errors. Then, the separation index for the first group was 6.52, for the second group was 7.06, and for the third group was 5.91, suggesting that they were divided respectively into six, seven, and five strata of severity among themselves in the group. The separation reliability of all the groups achieved more than 0.95, indicating that the separation statistics provided for all the groups are highly reliable.

**TABLE 10:** Teachers’ differences in severity based on teaching experience.

Group	Total score	Total count	Observed average	Measure	Model SE
1–10 years of teaching experience	6254	2418	2.6	−0.10	0.03
11–20 years of teaching experience	5948	2323	2.6	−0.07	0.03
More than 20 years of teaching experience	5239	2087	2.5	0.02	0.03
Mean	5813.7	2276	2.6	−0.05	0.3
SD	520.7	170.4	0.0	0.06	0.00

Model, fixed (all same) Chi-square: 7.3, *df*: 2, significance (probability): 0.00.

**TABLE 11:** Rater facet report based on teaching experience.

Group	No experience	1–3 years of experience	4–6 years of experience
χ^2^	1125.2	1462.8	1078.4
*df*	49	55	57
Significance	0.00	0.00	0.00
Separation ratio	4.64	5.05	8.18
Separation index	6.52	7.06	5.91
Separation reliability	0.96	0.96	0.95

## Discussion

This study explored the differences in rating quality when teachers are grouped in different experience backgrounds in terms of their rating, training, and teaching experiences. The study aimed to determine whether those experiences can lead to teachers using different severity or leniency levels when assessing candidates’ answers. A rating system was developed in this study to collect the required data in the form of score marks in a speaking assessment of lower secondary school in Malaysia. The process of validity to ensure the suitability of the study in the Malaysian context was performed with the assistance of five expert panels by calculated using CVR. It shows that the items reflect the measurement aspect and fulfill the content validity. Then, the assumptions of the Rasch model were fulfilled before the analysis was performed. The data were first analyzed to ensure there were no missing data. For reliability and validity, item fit analysis was run, and if the infit values were within the acceptable range, it showed the items fit with the measurement. The analysis of item separation showed that the items used in the study were separated into 9 difficulty strata and able to discriminate into 12 levels of ability. This portrays the idea that the items are able to be distinguished by the ability of the person and the items difficulty. The scales used in the current study were also proved to be valid as they fulfilled all the pre-requisite criteria. It can be seen by the fitness of the items by MNSQ. Besides, the instrument is reliable to be used in terms of the suitability and consistency of the rating scale.

The analysis of the study has yielded severity parameter of the teachers through the calibration of all the assessment elements in the study, namely teachers, candidates, items, and domains. A comparison of teachers’ severity levels has shown that there is the absence of uniformity in their severity when assessing the candidates, which is needed before performing MFRM analysis (Huang et al., 2014). It is also known as a stochastic rating, which refers to the existence of a variety of rating quality at least between two raters (Linacre, 1994). Based on the severity parameter, the teachers were divided into three groups that are severe teachers, lenient teachers, and average teachers. Average teachers are those who rate the candidates with the appropriate severity level and do not create any erratic scores. Severe teachers were too harsh and strict when assessing candidates, while lenient teachers tended to give easy marks to candidates. These two groups of teachers are undesirable in the operational rating process and negatively impact the assessment system. A significant impact on candidates is deteriorating their motivation (Cummings et al., 2014). For instance, candidates with low ability level receive high marks from lenient teachers rendering them not to put more effort to strive in the future because they managed to achieve high marks even though their answers were not of high quality. On the other hand, high-ability candidates receive low marks from severe teachers. Consequently, the situation will demotivate them as it is difficult for them to get marks even though they managed to provide high-quality answers. As for teachers, their inability to rate with an acceptable severity level can downgrade their professionalism (Peabody and Wind, 2019) because psychometrically sound marks are not awarded to candidates when they are too severe or too lenient.

Teachers’ severity levels were then compared based on their experience background in terms of rating, teaching, and training. The significance of the differences was determined and further analyzed through the chi-square test. A significant difference was found when teachers were compared based on their rating experience. This is in line with social constructivism theory that outlines one’s attitude depends on how they use their experiences. This result has suggested that teachers’ experience in assessing high-stakes assessment specifically PT3 impacts how they assess their students in the classroom, including classroom-based assessment. A significant difference was also discovered when severity was compared based on teachers’ teaching experience. Interestingly, when teaching experience increases, their severity levels tend to decrease. Other studies have also reported that raters with more teaching experience become more lenient (Hsieh, 2011; Kang, 2012; Weilie, 2018). Perhaps, teachers with less experience rate candidates with high severity level because they are more zealous and idealistic in preparing the candidates for examination (Ro, 2019). In fact, they were also reported to have the capability to follow standardization procedures, which affected their way of assessing students in the classroom context (Rappleye and Komatsu, 2018). Thus, their severity level is something that cannot be compromised, and candidates need to be penalized for each mistake they make.

On the other hand, more experienced teachers deployed flexibility, which affected their severity because they understood candidates’ language development (Lumley and Mcnamara, 1995; Weigle, 1998). Their experience has made them become more realistic and appreciate candidates’ variability than focusing on achieving standardizing in assessment. It may be due to their experience of the washback effect of the assessment system on teaching and assessment process in the classroom context (Turner, 2006). Indeed, more experienced teachers can better understand the effect as they have seen many changes in the education system. Thus, they may have developed the idea of giving opportunities to candidates in classroom assessment as long as follow-up intervention is executed because the real battle is in the high-stakes assessment.

When compared based on teachers’ experience in attending rater training, no significant difference was found. It contradicts the majority of previous studies that reported that training had made differences in teachers’ rating quality (Fahim and Bijani, 2011; Tajeddin and Alemi, 2014; Kim, 2015; Attali, 2016; Davis, 2016; Duijm et al., 2017; Seker, 2018; Kang et al., 2019). In fact, training is among the important strategies to reduce rater invariability among raters (Fahim and Bijani, 2011). Additionally, when novice raters attended training, their inter-rater and intra-rater reliability increased. However, the current study offered a new finding as teachers, regardless of their status of training experience, rated the candidates’ answers with statistically indifferent severity levels. The severity levels of both groups were below zero logits. This finding has suggested that the PT3 training that some of the teachers have attended did not manage to make them a better rater as compared to the teachers who did not attend the training. Also, the training on high-stake assessment scoring did not give an impact on how they carry out an assessment in the classroom context (Kang et al., 2019). This is relevant because assessing students in the classroom is different than assessing candidates in high-stakes assessment, during which standardization is strictly applied. Thus, teachers are monitored to rate candidates without the interference of other irrelevant factors, especially their personal background. However, when teachers are involved in assessing candidates not in a high-stake setting, they are not restricted to standardization procedures and are given the freedom to be themselves. This is even truer when assessing speaking skill because teachers need to finalize candidates’ marks on the spur of the moment (Nyroos and Sandlund, 2014). Hence, teachers tend not to sustain the standardization element when awarding marks to candidates (Sundqvist et al., 2018). In such a situation, they let themselves use their personal identity and personality even though training has been given (Seker, 2018; Bijani, 2019). That is why literature has also discovered that rater training managed to enhance one’s rating consistency but not homogeneity and rater agreement (Eckes, 2015). This research also encourages and supports the recommendations by Esfandiari and Myford (2013) to explore english as a foreign language (EFL) settings and highlight teachers as assessors.

## Implication and conclusion

The findings of the current study have given important implications to the arena of assessment, especially in the Malaysian lower secondary school settings. Rater training for teachers needs to be revised by considering teachers’ real needs. This may include introducing the potential of rating invariability, especially in terms of how one may be inclined to use their own personal experience when judging candidates’ answers. Teachers should be made aware of the tendency for one to become a severe and lenient rater when they let their personal experience to influence the marks they give to candidates. Hence, training should offer teachers the discussion about differential severity, halo effect, central tendency, and randomness. Apart from that, teachers should be given the opportunities to practice marking candidates’ answers in training using answer samples and then let them analyze their rating quality. The choice of answer samples needs to be varied in terms of candidates’ performance in each item and domain. Also, MFRM should be used more widely in training to determine the extent to which teachers can produce a sound rating. The rich information from the analysis can be used to further enhance teachers’ rating skills and reduce the existence of rater bias. The systematic rating system developed in this research can be used as it is less costly and practical in the operational setting as it does not require all teachers to rate the answers of all candidates. In fact, the analysis offers more rich information as compared to the practice of moderation that usually takes place after rating procedures, which can only give input on the agreement among raters.

The study has limitations in terms of the method used as it did not include any qualitative element due to the shortage of time and resources. Also, the MFRM analysis using the data collected in the form of teachers’ ratings to the candidates has already offered rich information about the teachers’ rating quality. On top of that, the analysis only focused on the rater facet, and rater bias between raters and other facet such as candidates and items were not thoroughly analyzed. Rater bias is connected to rating quality, but it needs to be discussed on its own because it entails a huge discussion and in-depth explanations. It discusses whether there is erratic interaction between facets and the extent to which it affects the objectivity of the assessment system.

Future studies should endeavor in seeking the potential of rater bias happening between teachers and other assessment elements. This would enable to diagnose how bias may influence candidates’ marks and disadvantage some groups of candidates. Future studies are necessary to ascertain whether the assessment in our education system is on track in realizing the aspiration of offering quality assessment to all students. This study discovers that teachers’ ratings and teaching experience make teachers different in terms of their rating quality. Specifically, teachers with more rating experience rate candidates with the highest severity level, while teachers with the least teaching experience rate with the lowest severity level. Whereas training experience did not bring any difference to teachers’ rating quality. The findings suggest that one’s teaching and rating experience may affect the quality of marks that candidates receive. Therefore, these two factors must be considered when teachers are assigned to mark answers. This study gives information to the policy makers on the current state of our teachers in the assessment field. The contribution of this study to the literature, especially in the Malaysian setting, is important as it determines the impact of different experience backgrounds on teachers’ rating quality in speaking assessment.

## Data availability statement

The original contributions presented in this study are included in the article/supplementary material, further inquiries can be directed to the corresponding author.

## Ethics statement

The studies involving human participants were reviewed and approved by the Educational Planning and Policy Research Division (iaitu Bahagian Perancangan dan Penyelidikan Dasar Pendidikan), Ministry of Education (MOE). Written informed consent for participation was not required for this study in accordance with the national legislation and the institutional requirements.

## Author contributions

MMN and MMM: conceptualization, validation, resources, and data curation. MMN: methodology, software, formal analysis, investigation, writing—original draft preparation, and project administration. MMM: writing—review and editing, visualization, supervision, and funding acquisition. Both authors have read and agreed to the published version of the manuscript.

## Conflict of interest

The authors declare that the research was conducted in the absence of any commercial or financial relationships that could be construed as a potential conflict of interest.

## Publisher’s note

All claims expressed in this article are solely those of the authors and do not necessarily represent those of their affiliated organizations, or those of the publisher, the editors and the reviewers. Any product that may be evaluated in this article, or claim that may be made by its manufacturer, is not guaranteed or endorsed by the publisher.
